# The performance of practitioners conducting facial comparisons on images of children across age

**DOI:** 10.1371/journal.pone.0225298

**Published:** 2019-11-19

**Authors:** Dana Michalski, Rebecca Heyer, Carolyn Semmler

**Affiliations:** 1 Defence Science and Technology Group, Edinburgh, South Australia, Australia; 2 School of Psychology, University of Adelaide, Adelaide, South Australia, Australia; Ulm University, GERMANY

## Abstract

Determining the identity of children is critical to aid in the fight against child exploitation, as well as for passport control and visa issuance purposes. Facial image comparison is one method that may be used to determine identity. Due to the substantial amount of facial growth that occurs in childhood, it is critical to understand facial image comparison performance across both chronological age (the age of the child), and age variation (the age difference between images). In this study we examined the performance of 120 facial comparison practitioners from a government agency on a dataset of 23,760 image pairs selected from the agency’s own database of controlled, operational images. Each chronological age in childhood (0–17 years) and age variations ranging from 0–10 years were examined. Practitioner performance was found to vary considerably across childhood, and depended on whether the pairs were mated (same child) or non-mated (different child). Overall, practitioners were more accurate and confident with image pairs containing older children, and also more accurate and confident with smaller age variations. Chronological age impacted on accuracy with mated pairs, but age variation did not. In contrast, both age and age variation impacted on accuracy with non-mated pairs. These differences in performance show that changes in the face throughout childhood have a significant impact on practitioner performance. We propose that improvements in accuracy may be achievable with a better understanding of which facial features are most appropriate to compare across childhood, and adjusting training and development programs accordingly.

## Introduction

Facial image comparison is used every day to assist in determining identity. Facial comparison practitioners may make these decisions with or without the assistance of facial recognition technology [1]. While most facial comparison practitioners work predominantly with images of adults, many are also required to work with images of children [2]. Visa and passport processing officers, analysts working in anti-child exploitation task forces, and police investigating cases of missing children, may all need to make decisions based on images of children. However facial comparison ability is likely to be impacted due to the age of the children in images and the age variation between images being compared, as a result of facial growth and development throughout childhood. Depending on the agency’s business processes, the repercussions for making the wrong decision can lead to anything from a minor inconvenience to a serious threat to life, and this can differ depending on whether the images being compared are mated (same person) or non-mated (different people). For example, from a passport renewal context, if approval of a passport is delayed because there is uncertainty as to whether the renewal image is the same person as that in a previous passport, it can be an inconvenience for that passport holder to send in more images to prove they are who they claim to be (i.e., mated pair). If two images are believed to be the same person and they are in fact different people (i.e., non-mated pair), this could result in, for example, a kidnapped child being taken out of the country and exploited in some way. Typically, in operational applications, the critical need is to ensure that people who are not who they claim to be are accurately identified. Despite the consequences of their decisions, facial comparison practitioners need to be as accurate as possible with both mated and non-mated images, and are likely to employ different strategies to achieve these competing aims [3].

Practitioners across all levels have typically received some formal and/or on-the-job training to conduct this important task [2, 4–6], however error rates do vary [7–10]. Unfortunately most of this training, as well as the previous research conducted in the facial comparison space, has focused almost entirely on images of adults [3–5, 7, 9, 11]. Given how much the face changes during childhood, it is unlikely that results of facial comparison studies using images of adults can be extrapolated to infer performance with images of children.

### Previous research

Although many government agencies conduct facial image comparisons of children with age variations often ranging from 0–10 years [2], research that has examined performance in this area is scant. Only one previous study has employed a comprehensive evaluation across age in childhood using operational images, however this study evaluated the performance of commercial facial recognition algorithms, not practitioners [12].

Understanding practitioner performance is vital. A previous study has been conducted to examine practitioner performance with image pairs of children compared to image pairs of adults [2]. A total of 200 pairs containing controlled passport style images were used (100 children, 100 adults). The results showed practitioner performance was poorer on image pairs of children (74%) compared to image pairs of adults (92%). Novices have also shown to be poorer with images of children (39%) than with images of adults (45%) on a one-to eight facial comparison task [13]. Further research with images of children is required to determine to what extent age and age variation is impacting on this performance.

Research that has examined the younger ages in childhood evaluated novice performance on image pairs where both images depicted either infants (< 1 year old) or an infant and a 4 or 5 year old [14]. The research found that accuracy with image pairs of infants was 56% whereas accuracy with image pairs containing an infant and 4 or 5 year old was 52%. Although this shows the impact age variation may have, the results are based on novices, being presented with cropped and greyscale images which do not reflect typical operational applications. Hence, performance may differ to that in the real-world.

Another study evaluated the performance of 76 participants (ranging from novices to experts) conducting facial comparisons with predominantly uncontrolled images of children [15]. Participants were exposed to 20 one-to-one trials and 10 one-to-ten trials. Age variation ranged from 0–5 years, but the majority were between 0–3 years (with no images at a 4 year age variation and only one at 5 years). Overall performance was 65.79% for one-to-one trials and 50.72% for one-to-ten trials. The need for a much larger study to be conducted using images across the childhood age span was highlighted, and the authors recommended using controlled images so that the impact of age could be appropriately evaluated in the absence of other variables that are known to impact on performance (such as image quality, pose, and expression).

Our study sought to address the limitations of previous research by incorporating operational images, a more operationally realistic methodology, facial comparison practitioners as participants, and examining performance by individual age in childhood, rather than considering children as one overall group. We tested practitioner accuracy using an experimental application we developed to run on the practitioner’s desktop computers, where participants had to decide whether images in a pair belonged to the same person (mated image pair) or different people (non-mated image pair). In the operational setting, images are presented to a practitioner for a reason; namely that a facial recognition algorithm has deemed them to score above a given threshold of similarity. For example, two images of the same child taken some time apart, would be compared as part of the passport application verification process. A fraudulent passport application may present two different, but similar looking children for verification [16]. For this reason, a commercial facial recognition algorithm (FaceVACS B8) supplied to us by Cognitec Systems GmbH was used during the image selection process for our study, rather than relying on randomly selected images.

The overarching aim of our study was to determine the impact of age and age variation on facial comparison practitioner performance with images of children. Due to potential differences in the repercussions for making the wrong decision in operational contexts, dependent on whether the image pair is mated or non-mated, performance by image pair type was also examined.

## Ethics statement

**Fig 1:**
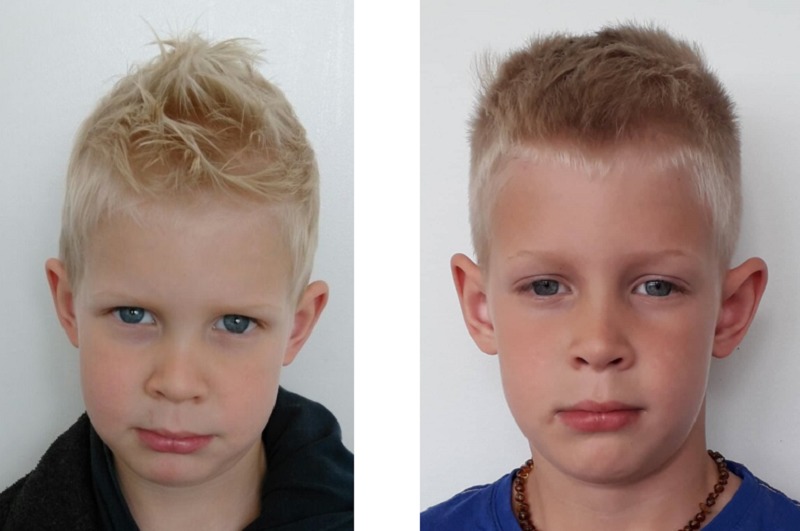
An example of the type of image pairs presented. Images are for illustration purposes only, and depict a mated pair (the same individual). The parents of the individual pictured in Fig 1 have given written informed consent (as outlined in PLOS consent form) for their images to be published.

This study was approved by the Human Research Ethics Committees at the Defence Science and Technology Group (approval number NSID 03/13) and the University of Adelaide (approval number 13/97). As the study was computer based, consent was obtained electronically at the beginning of the study. Written informed parental consent was provided for the child depicted in Fig 1 (as outlined in the PLOS consent form) to publish their images.

## Experiment

We examined practitioner performance on image pairs of children at every age (0–17 years) and age variations ranging from 0–10 years. A 10 year age variation was selected as this was available in the database, and because many agencies have reported a need to make facial comparisons on images of children with up to a 10 year age variation [2]. The methodology was also unique in that image selection involved a commercial facial recognition algorithm supplied to us by Cognitec Systems GmbH to return the highest scoring non-mated images. An image was then selected that followed strict inclusion criteria, including similar appearance and image quality (Table 1). Given that this was a unique opportunity to test 120 practitioners from a government agency, our aim was to select image pairs that may be more realistic in operational settings. Achieving an ecologically relevant stimulus set was extremely resource intensive, but was considered an important goal given the participating agency’s interest in understanding performance in this important task.

**Table 1:** Inclusion criteria and justification for selection of image pairs.

Inclusion criteria	Justification
No to minimal pose, illumination, or expression issues	To ensure the age variable was being tested in isolation as much as possible from other variables known to impact on performance and to keep consistent with ID document standards.
No blur	Blur may impact on performance but can also cause eye strain.
No occlusions	No occlusions on the face, such as glasses, that could be removed at the time of acquisition.
Neutral background	To remove distractions in the background (such as a mother in the background of an image of a young child).
Similar image quality between images in a pair	To reduce the possibility of image quality impacting on performance, particularly over longer age variations on mated pairs.
Loosely similar appearance e.g., ethnicity and gender	To ensure image pairs were not too easy and to keep consistency between and within pairs.

## Method

### Participants

Participants were 120 facial comparison practitioners (90 Female; Mean age = 41.49, SD = 10.65) from a government agency. Practitioners had experience in making facial comparison decisions ranging from 1 month to 36 years. The majority of participants (84.17%) were Caucasian. Practitioners were from the facial reviewer level as defined by the Facial Identification Scientific Working Group (FISWG) [6].

### Stimuli

Stimuli used in this experiment were controlled operational images where consent to use the images for research purposes had been obtained during the processing of the application. The database contained several million, front-on, coloured facial images with neutral expression. Image quality standards ensured variables such as pose, illumination, and expression were controlled as much as possible. The ages of people in the database ranged from less than one month to 105 years of age. The number of facial images per person in the database ranged from 1–14 images. For those with multiple images, the age variation ranged from one month to 13 years. No identifying information, such as name or address of the person in each image, was supplied with the images for this research. Instead, a random ID number was provided to link multiple images of the same person. Metadata supplied for each image included the month and year of birth of the person, the month and year when the image was supplied, and gender. No information regarding ethnicity was available, however, the ethnicity of the people in the database reflected the Australian population.

We manually selected 23,760 image pairs for this study using a software package called Comparer, developed by our team for image selection purposes [17]. Comparer is used to assist with the manual selection of images for experiments, as well as to assist agencies to check data integrity issues. It enables images, in this case image pairs, to be displayed next to each other for comparison and assessment for inclusion in a study. Selecting a check box next to a pair adds that pair to a list for further review, automatically populating a spreadsheet of image names for inclusion in a study, and assisting with retrieval of those images from a larger dataset. For every age in childhood (0–17 years) and every age variation in years ranging from 0–10 years, we selected 120 image pairs to ensure we had a balance of male and female, mated and non-mated pairs in order to generate the data we would need to conduct our analyses. This resulted in 198 different categories (198 categories x 120 pairs per category = 23,760 pairs). We used a different selection procedure for the mated and non-mated pairs.

#### Mated image pair selection

We required 11,880 mated image pairs for this study, which included 60 mated image pairs (30 male, 30 female) at every age in childhood (0–17 years) and every age variation ranging from 0–10 years. The IDs of all appropriate mated pairs of children from the database were separated in Microsoft Excel^™^ by the 198 required categories and gender. We used a random number generator in Microsoft Excel^™^ to randomly order these pairs by category and gender, and manually inspected them with the assistance of the Comparer software, to ensure they met the inclusion criteria (Table 1). If an image pair did not fulfil the criteria, the pair was discarded and the next appropriate image pair was selected. This process continued until all 30 female mated image pairs and all 30 male mated image pairs were selected for each of the 198 categories.

#### Non-mated image pair selection

We required 11,880 non-mated image pairs for this study. Five steps were taken to select the non-mated pairs:

1. one commercial facial recognition algorithm supplied to us by Cognitec Systems GmbH was used to conduct a one-to-many search on each of the 11,880 youngest age images used in the mated pairs (the same age variation and gender was used from those mated pairs);
2. the top 30 highest scoring non-mated images for each of the 11,880 images were returned;
3. the 30 image pairs were sorted from highest scoring to lowest scoring for each of the 11,880 images;
4. the first image pair from the 30 pairs that fulfilled the inclusion criteria (Table 1) was manually selected; and
5. step 4 was repeated another 11,879 times.

In summary, the youngest age image from every mated pair was used as the youngest age image in every non-mated image pair. The same age variation was used for the mated pairs as the corresponding non-mated pairs. The 23,760 image pairs were then manually re-examined with the assistance of the Comparer software to ensure they met the inclusion criteria (Table 1), and ID numbers were checked in Microsoft Excel^™^ to ensure that all other images were only ever used once.

Once this process was complete, using the Comparer software, two independent judges manually screened the 23,760 image pairs. This was to ensure consistency in image quality and consistency within the image pairs, particularly as the age variation increased, because image quality standards within the agency had changed over the 10-year period. Poorer performance on mated pairs with longer age variations could have been due to poorer image quality, rather than the time that had elapsed between images if this was not accounted for. This was not necessarily an issue with non-mated pairs as the image pairs could have been taken in the same year (as long as the appropriate age variation was selected). The judges were provided with the inclusion criteria (Table 1) to ensure that each image pair complied. One final check over the 23,760 image pairs was then conducted, using the Comparer software, to ensure consistency throughout the dataset. This image selection process took four months.

#### Experimental application

The experimental application was designed based on computer screen layouts used by facial comparison practitioners in their day-to-day work, observed during an earlier study [2]. The application included a login screen, an information screen, a series of demographic questions, instructions for the study, the 198 face matching trials, and a final screen containing a post-study question.

## Procedure

Approval by higher management was sought within the participating agency, followed by section managers, prior to seeking interest from facial comparison practitioners. Practitioners were sent an email the week prior to the study opening to give them an opportunity to plan when they would set aside a block of time of around one hour to conduct the study. This was followed a week later by another email which included a link to the study which was available for them to access on their own desktop computer via the agency’s intranet, along with a unique ID and password. The email also informed practitioners that they could withdraw from the study at any time, that they were to work on the study alone, and that their individual results would not be provided to management.

Practitioners answered a range of demographic questions and were then randomly presented with 198 image pairs (99 mated, 99 non-mated) and asked to decide if the pairs were of the ‘same’ person or ‘different’ people and rate their confidence on the provided scale (0–100% scale with selection options at 10% increments). Practitioners were instructed to work as quickly and accurately as possible, but were not deadlined. Fig 1 provides an example of what these image pairs looked like.

Each of the 120 practitioners was presented with a unique set of 198 image pairs. Thus, each practitioner viewed a different set of images, as would typically be expected in the operational setting where a single practitioner would ordinarily view their own set of images. This approach was chosen as our focus was on performance at different ages in childhood over different age variations, rather than individual differences amongst practitioners. The results, therefore, represent group level performance across the age ranges.

Following the 198 one-to-one trials, participants were asked a final question: “Please comment on any methods you used to help make a decision. For example, looking at the whole face overall, gut feeling, specific facial features etc.” This was to determine the strategies that practitioners claim to use when making these decisions.

## Results

**Fig 2:**
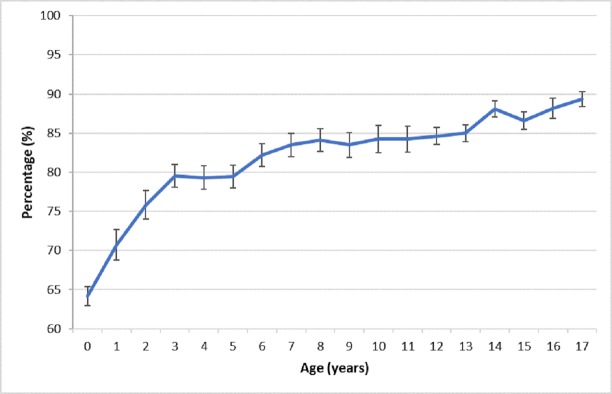
Overall accuracy across ages (%).

We used non-parametric tests for comparison of means and a critical alpha level of .05. Overall, accuracy was poorer earlier in childhood than later (Fig 2), likely due to younger children having less distinguishing facial features [18, 19].

We used heat map data matrices to summarise both accuracy and confidence by age and age variation, from both an overall and image pair type (mated and non-mated) perspective. The heat map data matrices are coloured so that green indicates the best performance, yellow the midpoint, and red the worst.

### Practitioner performance with images of children at each age and age variation

**Fig 3:**
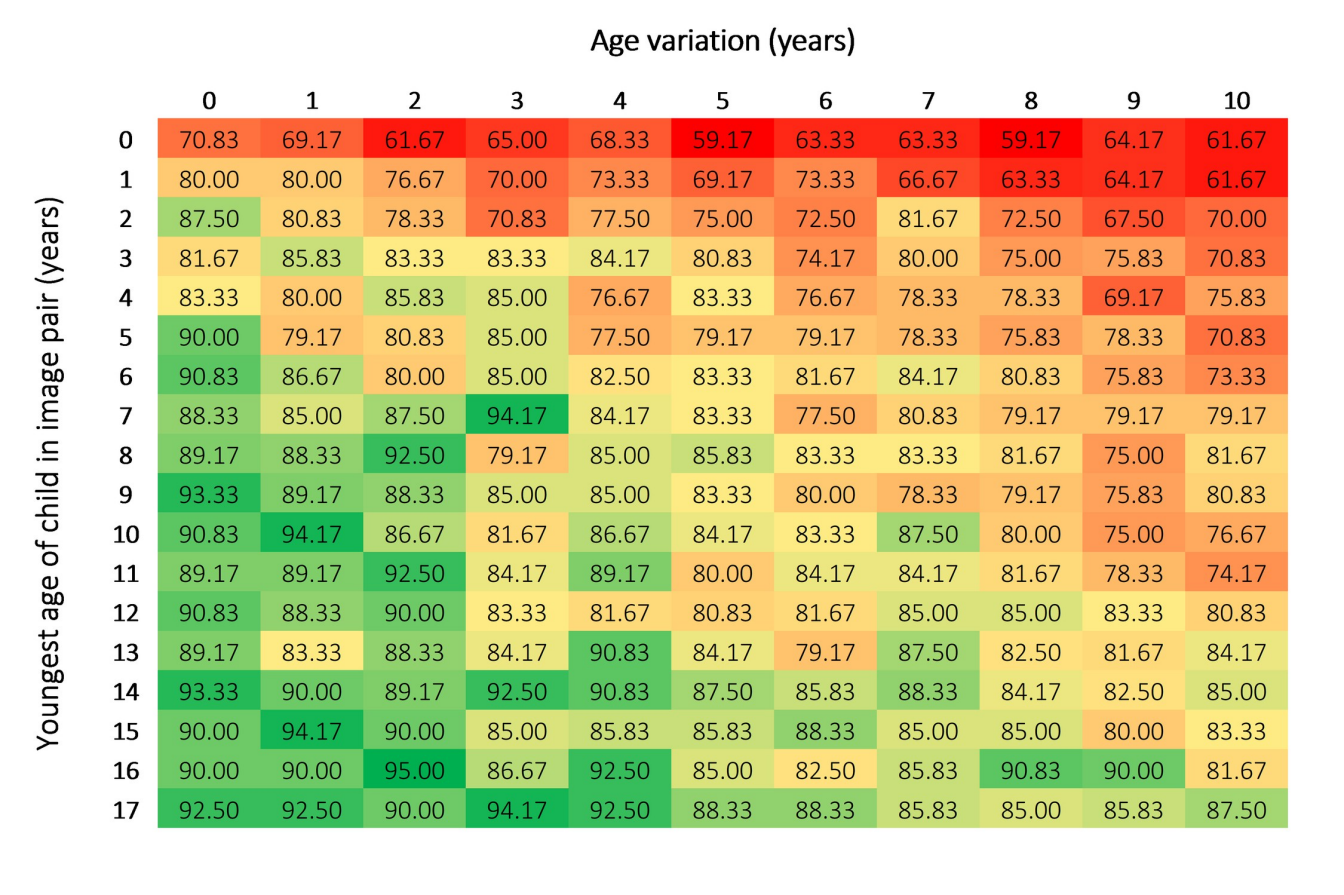
Overall accuracy for each age and age variation (%). Image pairs are grouped based on the age of the youngest child in an image pair and include age variations ranging from 0–10 years. For example, the top left group (with the value of 70.83%) represents a pair where the youngest child was aged 0 years and the age variation between the images was less than 12 months (e.g., a 0 year old and a 0 year old). The bottom right group (with the value of 87.50%) represents a pair where the youngest age was 17 years and the age variation was 10 years (e.g., a 17 year old and a 27 year old).

Overall accuracy ranged from 59.17% to 95% (M = 81.8%, Mdn = 83.3%, SD = 7.7; Fig 3). Patterns of performance can clearly be seen in the heat map data matrix. For example, overall accuracy was best in the lower left corner of the matrix indicating that facial comparison practitioners performed more accurately with images of children that were older and which had less age variation between images.

**Fig 4:**
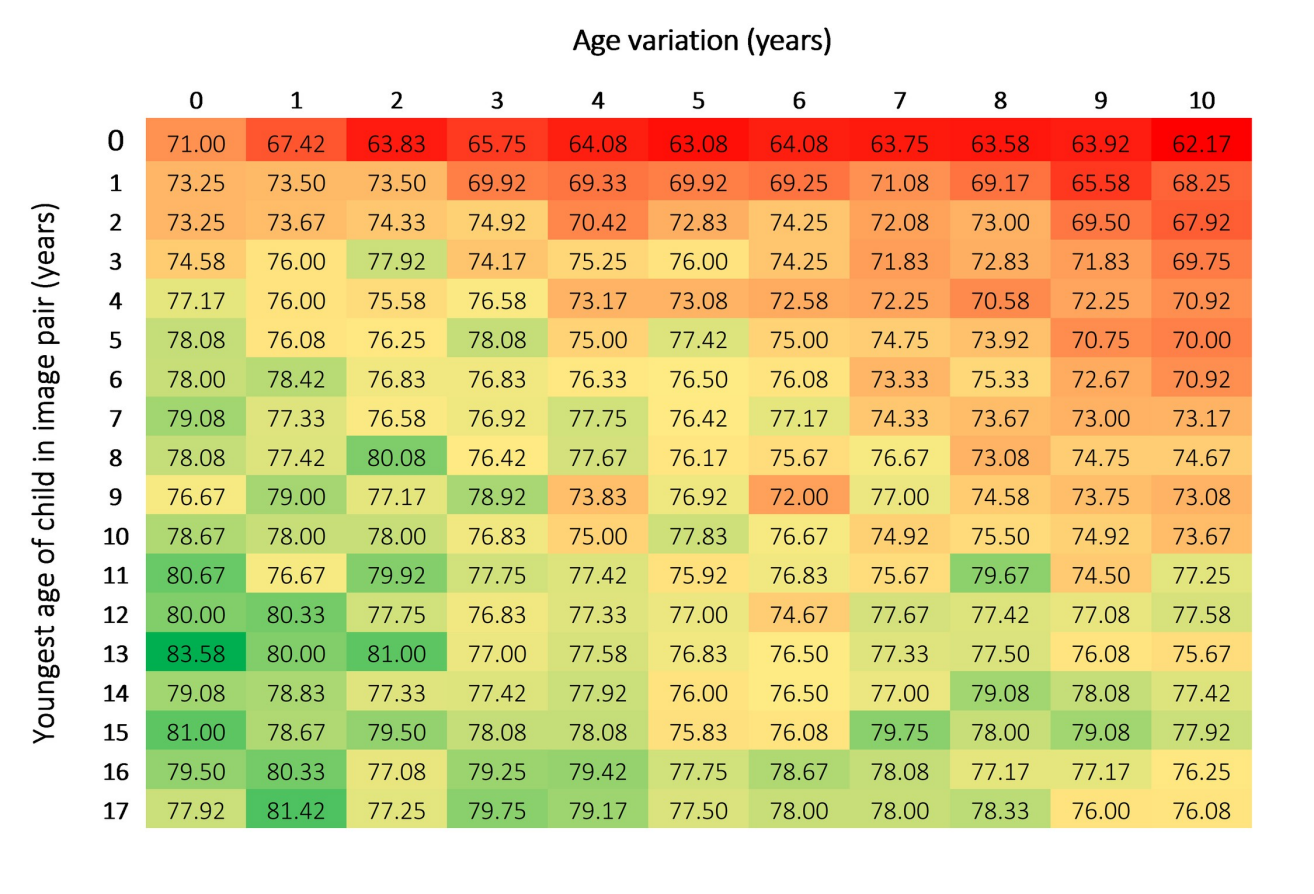
Overall confidence for each age and age variation (%).

Overall confidence ranged from 62.17% to 83.58% (M = 75.32%, Mdn = 76.5%, SD = 3.9; Fig 4). Trends in overall confidence over age and age variation were reasonably consistent with the overall accuracy data, in that the older the child and the shorter the age variation, the more confident the facial comparison practitioners were in their decisions.

### Practitioner performance with images of children at each age and age variation on mated and non-mated image pairs

**Fig 5:**
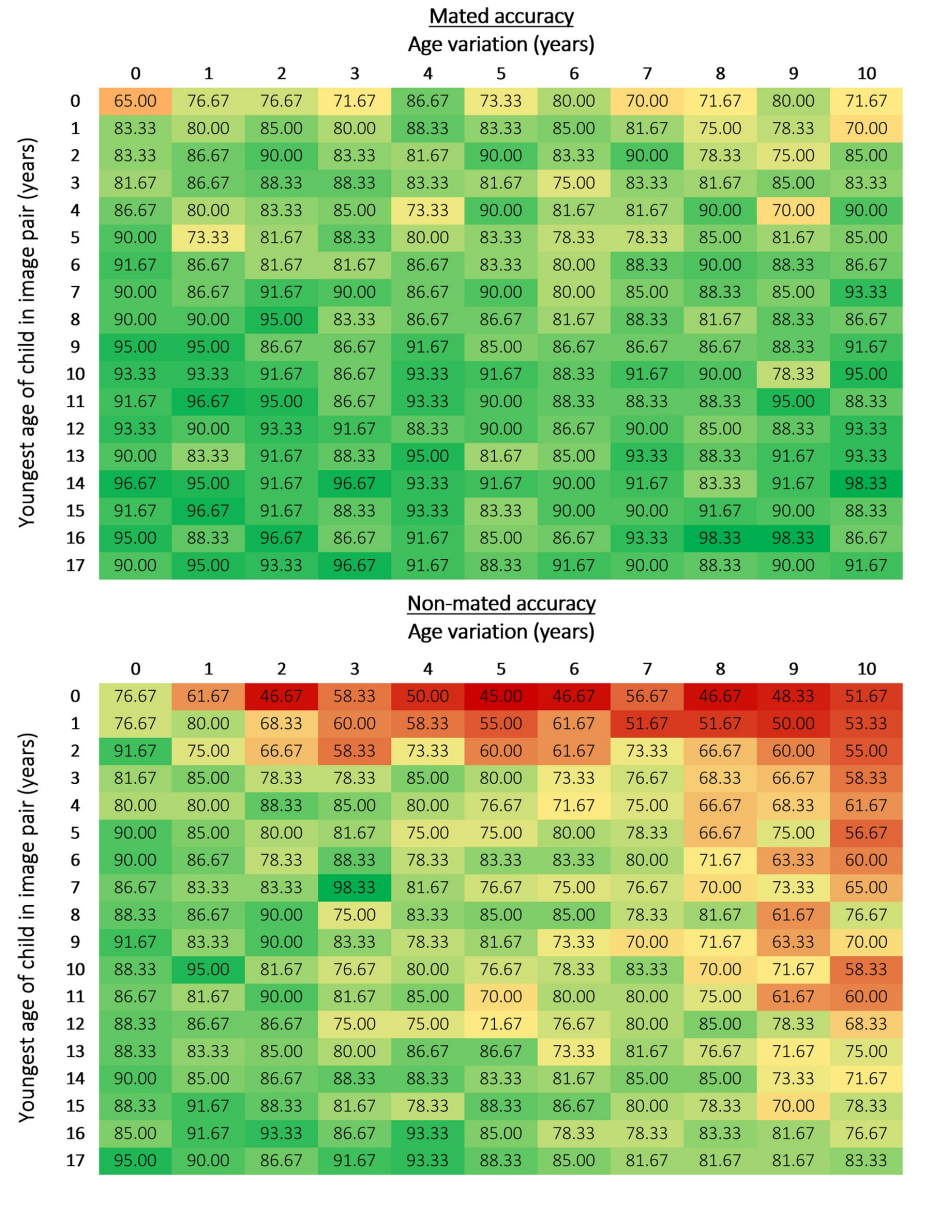
Accuracy for mated and non-mated image pairs at each age and age variation (%). The mated and non-mated heat map data matrices used the same colouring format rules to show how performance collectively varied based over the two pair types (i.e., lowest accuracy was coloured red and highest was coloured green, with yellow representing the midpoint of the highest and lowest scores).

Accuracy on mated pairs ranged from 65% to 98.33% (M = 86.97%, Mdn = 88.33%, SD = 6.21; Fig 5) and was impacted by age (χ^2^ (17) = 184.99, *p* < .001) but not age variation (χ^2^ (10) = 14.15, *p* = .166). Thus, the youngest age of a child in a mated image pair impacted performance, but the age variation between mated images in a pair did not.

Non-mated accuracy ranged from 45% to 98.33% (M = 76.66%, Mdn = 78.33%, SD = 11.41; Fig 5). In general, performance was poorer when an image pair contained an infant, regardless of age variation, and there was a noticeable decline in accuracy for age variations above 7 years on non-mated pairs. In contrast to mated pairs, practitioner performance for non-mated pairs was impacted by both age (χ^2^ (17) = 301.46, *p* < .001) and age variation (χ^2^ (10) = 199.81, *p* < .001), suggesting that both the age of the youngest child in an image pair and the age variation between the two images impacted decision making.

In summary, facial comparison practitioners were more accurate with mated image pairs (M = 86.97%, Mdn = 88.33%) than non-mated image pairs (M = 76.7%, Mdn = 78.3%; *z* = -10.67, *p* < .001, *r* = -.54).

**Fig 6:**
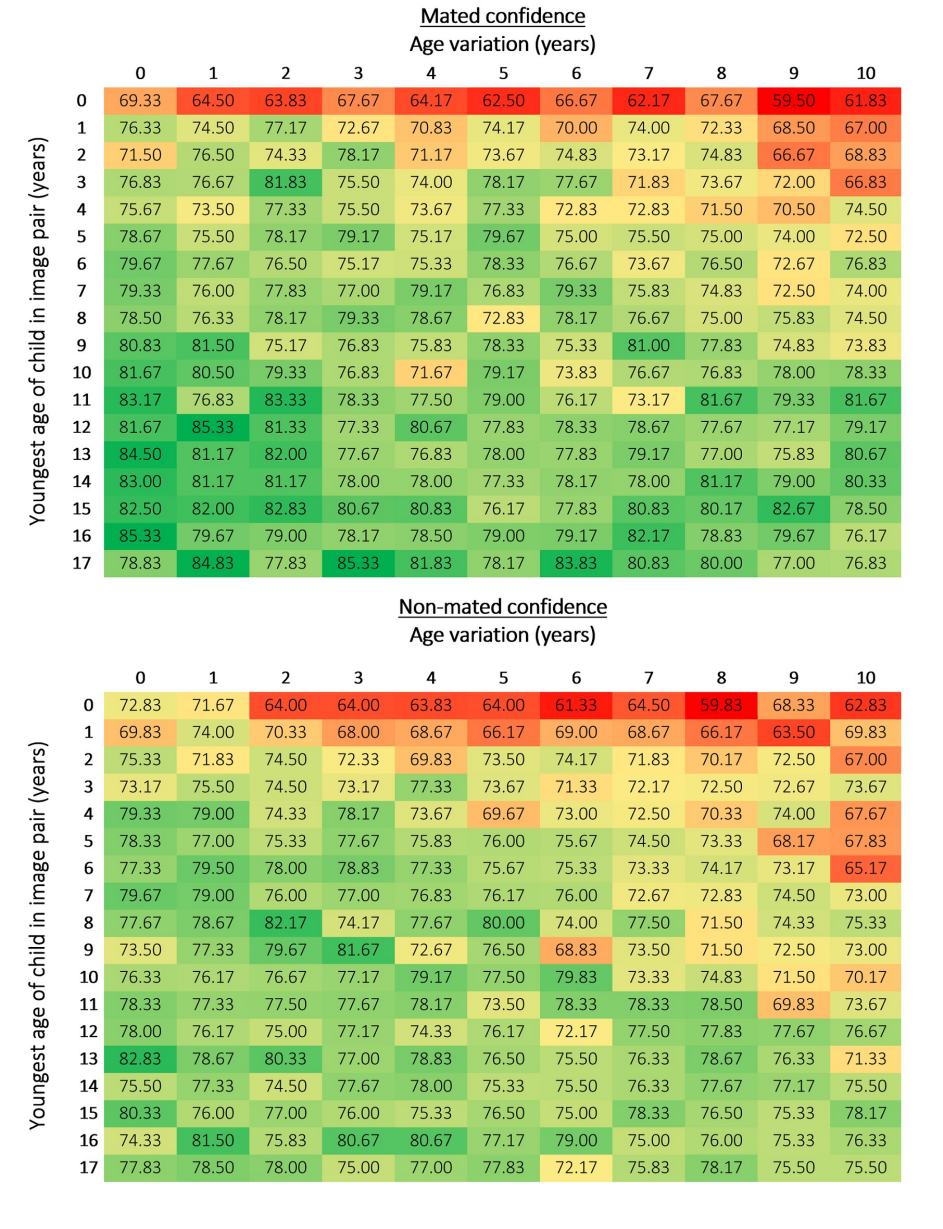
Confidence for mated and non-mated image pairs for each age and age variation (%). The mated and non-mated heat map data matrices used the same colouring format rules to show how confidence collectively varied based over the two pair types (i.e., lowest confidence was coloured red and highest was coloured green, with yellow representing the midpoint of the highest and lowest scores).

There was a similar pattern of results for confidence. Facial comparison practitioners were less confident when making decisions with non-mated image pairs (M = 74.61%, Mdn = 75.50%) than with mated image pairs (M = 76.55%, Mdn = 77.25%; *z* = -6.59, *p* < .001, *r* = -.33; Fig 6).

Confidence with mated image pairs ranged from 59.50% to 85.33% (M = 76.55%, Mdn = 77.25%, SD = 4.57). The older a child was in the youngest image, the more confident practitioners were in their decisions. Confidence on mated pairs significantly varied based on age (χ^2^ (17) = 400.48, p < .001) and age variation (χ^2^ (10) = 77.98, *p* < .001). In contrast, practitioner’s confidence with non-mated image pairs ranged from 59.83% to 82.83% (M = 74.61%, Mdn = 75.50%, SD = 4.14). Confidence varied based on both age (χ^2^ (17) = 266.41, *p* < .001) and age variation (χ^2^ (10) = 125.29, *p* < .001).

### Strategies adopted by practitioners to make facial comparison decisions

At the conclusion of the study we asked practitioners about what strategies they used and/or facial features they compared when making their facial comparison decisions. Answers to this question were categorised, so, for example, if a practitioner mentioned one or multiple components of the nose (e.g., nostrils, bridge), this was grouped into the ‘nose’ category and recorded once (as one practitioner mentioned components of the nose). The frequency of responses for each practitioner was calculated (Table 2).

**Table 2:** Strategies/features adopted to make facial comparison decisions.

Strategy/feature	Number of practitioners (%)
Ears e.g., shape, size, position	63 (52.50%)
Nose e.g., shape, nostrils, bridge	37 (30.83%)
Eyes e.g., shape, inner canthus, eyelid shape, outer area of eyes	37 (30.83%)
Mouth e.g., lip shape, size, gap between lips, cupids bow	28 (23.33%)
Whole face	26 (21.67%)
Gut feeling	21 (17.50%)
Markings e.g., freckles, moles, blemishes	21 (17.50%)
Individual facial features (not specified)	20 (16.67%)
Face shape	13 (10.83%)
Chin e.g., shape, distance from other features	7 (5.83%)
Eye pupil distance e.g., relative to each other and rest of face	6 (5.00%)
Hair e.g., hairline, hair patterns	6 (5.00%)
Jaw e.g., jawline and shape	4 (3.33%)
Eyebrows	2 (1.67%)
Forehead e.g., size, forehead-face ratio	2 (1.67%)
Philtrum	1 (0.83%)
6 FR points i.e., ears, eyes, nose, mouth, shape of face, facial marks	1 (0.83%)
Took into account pose, lighting, expression	1 (0.83%)

These results were not substantially different from a previous survey of facial comparison practitioners which asked them to list the facial features they used for making decisions on adult faces [4]. In that survey, the eyes, nose, ears and mouth were also listed as the most useful features, likely reflecting the training that these practitioners had received [5, 20]. However, strategies adopted amongst a relatively homogenous group (trained facial comparison practitioners from the same agency) varied considerably. Some practitioners started by viewing the whole face then breaking it down into facial features, if necessary. However, while some practitioners said they looked at the face as a whole for at least part of their strategy, others were insistent that they would not look at the face as a whole at all.

## Discussion

Our aim was to gather data at the finest level possible, rather than arbitrarily group children into age groups or age variations that may not be relevant to a wider group of end users of the research. Conducting this study at such a fine grained level enabled us to discern trends and patterns in the data over the 198 different categories in an easy to understand format (the heat map data matrices). Due to the substantial size of this study, the data can be rolled up to answer agency specific questions where necessary. Data were also separated into pair types (i.e., mated and non-mated) to enable end users to better understand performance differences so that appropriate mitigating strategies and/or training could be implemented where necessary.

### Practitioner performance with images of children across age and age variation

In general terms, as age increased, performance of practitioners improved, a finding consistent with anecdotes provided by facial comparison practitioners [2, 4] and results from previous facial comparison [13] and commercial facial recognition algorithm evaluations [2, 12, 21]. Even when the age variation was kept constant at 0 years, performance was worse for younger ages in childhood. From an operational perspective, these results highlight that even if age variation between images is less than a year, it is difficult to determine if younger children (i.e., infants and toddlers) are the same person or different people, compared to if the images contained a child at 5 years of age or older, when performance becomes more consistent. This has important implications for the use of passports and other identity documents with infants, and for law enforcement who may need to identify very young missing or exploited children.

Overall accuracy differed by up to 35.83% based on age and age variation. There was up to a 33% difference based on age alone (i.e., when the age variation was 0 years). Poorer performance at younger ages may be due to children having less discriminating facial features, but also due to the amount of facial change occurring early in life [18, 19, 22]. Previous research has found that age variations of up to five years with image pairs of children had minimal impact on overall performance compared to other variables that may have impacted on performance, such as image quality [15]. In this study, we controlled for image quality and still demonstrated the considerable impact that age variation between images has on performance.

In terms of confidence, practitioners were the least confident with pairs containing younger children and most confident when images were of older children, with shorter age variations. Practitioners tended to be slightly more accurate than confident at each age and age variation. The patterns in performance suggest that practitioners were generally aware of their abilities across the age-related conditions, although there were larger differences between accuracy and confidence for some individual categories. For example, practitioners were considerably under confident with image pairs at age 7 with a 3 year age variation (17.25% different between accuracy and confidence). More noticeable differences between accuracy and confidence were seen once the data was divided into pair type.

### Practitioner performance with images of children for each age and age variation on mated and non-mated image pairs

Practitioners were generally less accurate on non-mated image pairs. This is consistent with our expectation that non-mated pairs would be more difficult than mated pairs, and findings from previous research showing a difference between these two facial comparison tasks [3]. This difference may have been exacerbated because children have less discriminating facial features, making them more difficult to distinguish from one another [22].

Performance on non-mated pairs also decreased as the age variation increased. Practitioners appeared to change their decision threshold as the age variation increased. Previous work investigating the cognitive aspects of visual perception may provide a likely explanation [23]. It has been posited that ‘possible and permissible’ variations of appearance help to determine the boundaries between one face and another; and that our general knowledge of faces and how they change allows us to anticipate how they may appear with a different expression, or at another viewpoint [24]. We believe that practitioners used their general knowledge of how faces change to anticipate how they may appear at a different point in time. This becomes problematic when comparing non-mated image pairs of children over long age variations because what is believed to be ‘possible and permissible’ becomes a lot broader and, as a result, accuracy decreases. Further, intra-individual variation in changes to facial features cannot be easily learned without exposure to the population. It may be that more training on how the face actually changes across childhood would improve accuracy if practitioners were provided with information about what is morphologically ‘possible and permissible’.

Another reason for higher error rates among non-mated images may be prior work experience. Practitioners may be used to selecting ‘same’ as a default option since their work comprises comparisons of predominantly mated image pairs. Thus, they may only select ‘different’ if they can actually see something dissimilar between images in a pair that cannot be explained. It would be beneficial to conduct further research with novices, or a group of practitioners who are exposed to more non-mated than mated pairs, to see if that pattern of results is similar to those found here. If the results change with the base rate of presentation of non-mated pairs, as has been seen in other studies [25], it would show sensitivity to the variation rather than reliance on an assumption about the rare occurrence of non-mated pairs.

Accuracy for mated pairs was relatively stable across age variation. This may also be explained by practitioner’s perceptions as to what they believe is ‘possible and permissible’. For example, at shorter age variations, practitioners may see the similarities within the two images being compared, but as the age variation increases there is a shift in their decision criterion as they need to rely more on what they believe is ‘possible and permissible’. Once again, a better understanding of what facial features are most stable in childhood for comparison purposes would ensure that practitioners were basing their decisions on knowledge, rather than on assumptions.

Nevertheless, the accuracy rates of facial comparison practitioners with mated (89.87%) and non-mated (76.66%) images of children that were obtained here is promising. This is particularly important given the accuracy rates in past research [8, 15] and that image pairs in the current study included infants and toddlers, and age variations of up to 10 years.

**Fig 7:**
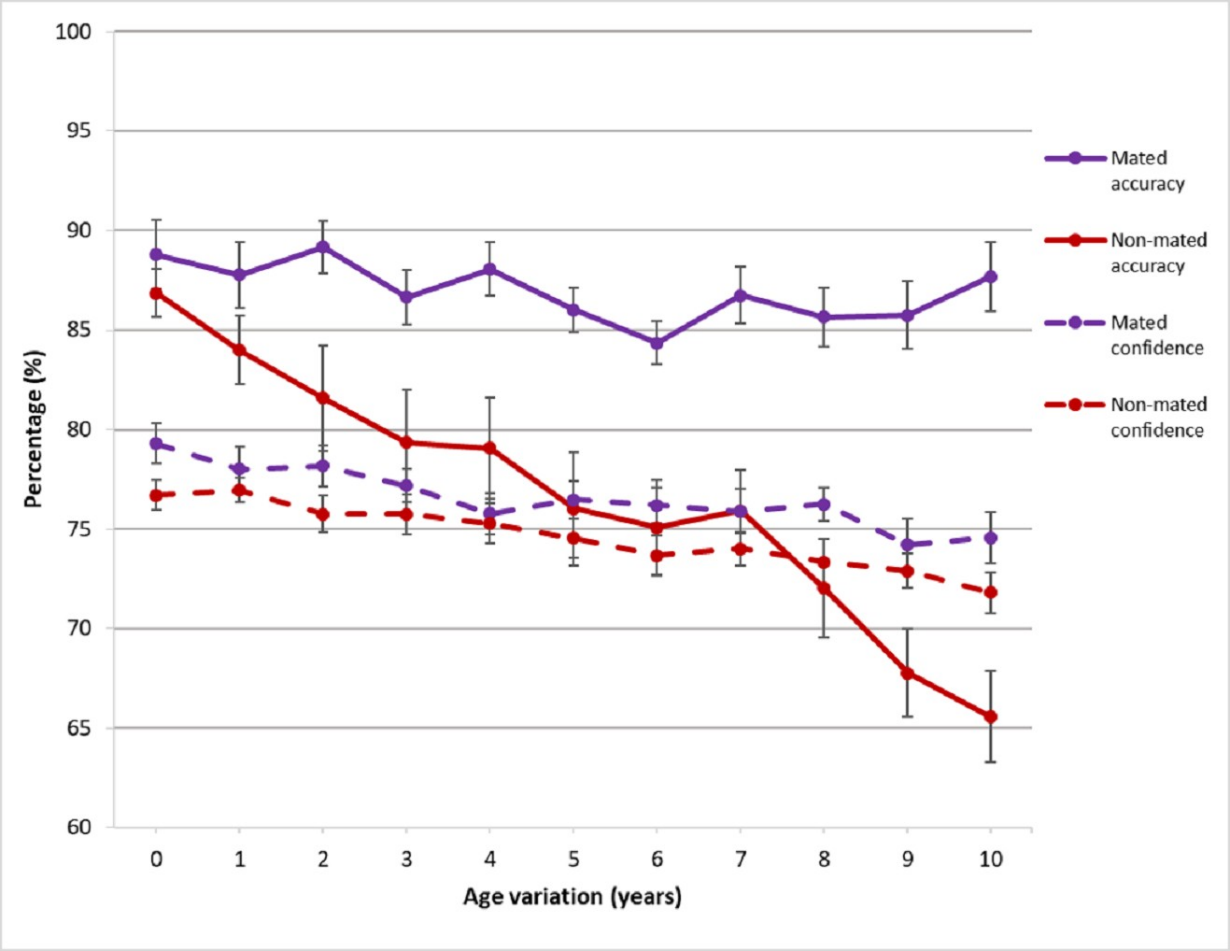
Accuracy and confidence based on pair type across age variation.

Confidence of practitioners remained relatively consistent with mated and non-mated pairs across age variation. Practitioners were under confident when making decisions on mated pairs (accuracy remained higher than confidence at each decision point). However, practitioner accuracy dropped on non-mated pairs after a 7 year age variation, with little change in confidence, suggesting a trend towards over confidence as the task got more difficult (Fig 7).

In summary, there was considerable variance in performance across childhood based on age and age variation. The diverse results over age and age variation suggest that agencies should exert caution when conducting facial comparisons with images of children, particularly at younger ages. Given that the images in this study were controlled (e.g., passport or visa style), it is likely that performance would degrade even further with uncontrolled images (e.g., social media style) over the same ages and age variations. We recommend further studies with less controlled images to confirm this.

### Strategies adopted by practitioners to make facial comparison decisions

Facial comparison practitioners typically reported using multiple strategies throughout the study to help inform their decisions. Although collecting information regarding strategies adopted by facial comparison practitioners is insightful, it is also subjective. Therefore, we recommend further research to empirically investigate whether practitioners are using the strategies they claim, and to correlate which strategies result in higher performance. This could be achieved by conducting an eye tracking study to objectively determine the strategies employed [26].

Facial comparison training in agencies typically involves a morphological approach that requires comparing individual facial features and forming conclusions [5, 27]. This approach is recommended by the Facial Identification Scientific Working Group (FISWG) over other methods such as photo-anthropometry [28]. However, a morphological approach is likely easier with images of adults compared to children as adult’s faces are more stable over time, whereas children’s faces are still developing. Furthermore, there are currently no international standards as to what specific facial features should be examined. FISWG are now addressing this issue for images of adults [29]. However, standards for adults are not likely to be relevant to inform standards regarding images of children. For example, the nose undergoes a growth spurt between the ages of 1–6 years and can change considerably during this time [30], not becoming recognisable in its adult morphology until around 8 years of age [22]. Therefore, the nose may only become a reliable facial feature to use at this time, and may cause errors if relied upon earlier in childhood. What seems certain is that the strategy a practitioner uses will need to change depending on whether images are of younger or older children [31]. We believe that detailed longitudinal morphological data at a broad population level is required to understand what facial features become stable the earliest in childhood so practitioners have a better chance of making accurate decisions by being able to identify similarities and differences in stable facial features.

## General discussion

Our study was the largest of its kind to date; involving 120 facial comparison practitioners from a government agency and 23,760 unique image pairs carefully selected from an operational database to understand facial comparison performance with images of children. The data we have generated can help end users, such as law enforcement and national security agencies, determine if facial comparison performance with images of children is reliable enough to continue with current business processes, or whether additional contingencies may need to be considered. These decisions will vary based on the agency, the application, and the feasibility of implementing new procedures.

The vast differences in performance with controlled images over age and age variation show that changes in the face throughout childhood have a significant impact on practitioner performance. Although practitioner performance in this study was better than previous studies, we believe improvements in accuracy may be achievable with a better understanding of which facial features are most appropriate to compare across childhood. It is highly likely that different facial features may be more appropriate to use when conducting facial comparisons of children than those currently taught for use with images of adults, or that different strategies may need to be adopted [31]. Identifying any stable facial features in childhood, through a better understanding of how the face changes during this time, would be particularly useful. Once validated, this information should be incorporated into facial comparison training and development programs in the future.

## References

[1] Prince J. To examine emerging police use of facial recognition systems and facial image comparison procedures. 2012; https://www.churchilltrust.com.au/media/fellows/2012_Prince_Jason.pdf.

[2] Michalski D. The impact of age-related variables on facial comparisons with images of children: Algorithm and practitioner performance [Doctoral Dissertation]: University of Adelaide; 2017.

[3] Megreya AM, Burton AM. Hits and false positives in face matching: A familiarity-based dissociation. Perception & psychophysics. 2007;69(7):1175–84. 18038955 10.3758/bf03193954

[4] Heyer R, MacLeod V, Carter L, Semmler C, Ma-Wyatt A. Profiling the facial comparison practitioner in Australia. DST Edinburgh, South Australia: DST-Group-GD-1030; 2017.

[5] Towler A, White D, Kemp RI. Evaluating the feature comparison strategy for forensic face identification. Journal of Experimental Psychology: Applied. 2017;23(1):47 10.1037/xap0000108 28045276

[6] FISWG. Recommendations for a training program in facial comparison Facial Identification Scientific Working Group; 2012 p. 1–6.

[7] Phillips PJ, Yates AN, Hu Y, Hahn CA, Noyes E, Jackson K, et al Face recognition accuracy of forensic examiners, superrecognizers, and face recognition algorithms. Proceedings of the National Academy of Sciences. 2018:201721355. 10.1073/pnas.1721355115 PMC6004481 29844174

[8] White D, Kemp RI, Jenkins R, Matheson M, Burton AM. Passport officers’ errors in face matching. PloS one. 2014;9(8):e103510 10.1371/journal.pone.0103510 25133682 PMC4136722

[9] White D, Phillips PJ, Hahn CA, Hill M, O'Toole AJ. Perceptual expertise in forensic facial image comparison. Proc Biol Sci. 2015;282(1814). Epub 2015/09/04. 10.1098/rspb.2015.1292 26336174 PMC4571699

[10] Heyer R, Semmler C, Hendrickson AT. Humans and algorithms for facial recognition: The effects of candidate list length and experience on performance. Journal of applied research in memory and cognition. 2018;7(4):597–609.

[11] Megreya AM, Sandford A, Burton AM. Matching face images taken on the same day or months apart: The limitations of photo ID. Applied Cognitive Psychology. 2013;27(6):700–6.

[12] Michalski D, Yiu SY, Malec C. The impact of age and threshold variation on facial recognition algorithm performance using images of children International Conference on Biometrics (ICB): IEEE; 2018 p. 217–24.

[13] White D, Dunn JD, Schmid AC, Kemp RI. Error rates in users of automatic face recognition software. PLoS One. 2015;10(10):e0139827 10.1371/journal.pone.0139827 26465631 PMC4605725

[14] Kramer RS, Mulgrew J, Reynolds MG. Unfamiliar face matching with photographs of infants and children. PeerJ. 2018;6:e5010 10.7717/peerj.5010 29910991 PMC6001712

[15] Ferguson EL. Facial identification of children: a test of automated facial recognition and manual facial comparison techniques on juvenile face images [Doctoral dissertation]: University of Dundee; 2015.

[16] Zhang S. Smuggling and trafficking in human beings: all roads lead to America Westport, CT: Praeger Publishers; 2007.

[17] Hole M, McLindin B, Hanton K, Malec C, Yiu SY, Hanly G. An overview of a DSTO developed human operator image comparison software tool—Comparer. Edinburgh, South Australia: DSTO-GD-0855; 2015.

[18] Kozak FK, Ospina JC, Cardenas MF. Characteristics of normal and abnormal postnatal craniofacial growth and development 2015 In: Cummings Pediatric Otolaryngology E-Book [Internet]. [55–80].

[19] Ricanek K, Mahalingam G, Albert AM, Bruegge RV, Fairhurst M. Human face ageing: a perspective analysis from anthropometry and biometrics. Book Chapter in Age Factors in Biometric Processing Edited by Michael Fairhurst. 2013.

[20] Towler A, White D, Kemp RI. Evaluating training methods for facial image comparison: The face shape strategy does not work. Perception. 2014;43(2–3):214–8. 10.1068/p7676 24919354

[21] Grother P, Ngan M. Face Recognition Vendor Test (FRVT)—Performance of face identification algorithms. NIST Interagency Report 8009; 2014.

[22] Wilkinson C. Juvenile facial reconstruction In: Wilkinson CR C, editor. Forensic facial reconstruction. Cambridge, New York: Cambridge University Press; 2012 p. 254–60.

[23] Vernon MD. A further study of visual perception: Cambridge University Press; 1952.

[24] Bruce V. Stability from variation: The case of face recognition the MD Vernon memorial lecture. The Quarterly Journal of Experimental Psychology. 1994;47(1):5–28. 10.1080/14640749408401141 8177963

[25] Stephens RG, Semmler C, Sauer JD. The effect of the proportion of mismatching trials and task orientation on the confidence–accuracy relationship in unfamiliar face matching. Journal of Experimental Psychology: Applied. 2017;23(3):336 10.1037/xap0000130 28805443

[26] Havard C. Eye movement strategies during face matching: University of Glasgow; 2007.

[27] Megreya AM. Feature-by-feature comparison and holistic processing in unfamiliar face matching. PeerJ. 2018;6:e4437 10.7717/peerj.4437 29503772 PMC5831152

[28] FISWG. Guidelines for facial comparison methods Facial Identification Scientific Working Group; 2012 p. 1–15.

[29] FISWG. Physical stability of facial features of adults Facial Identification Scientific Working Group; 2019 p. 1–16.

[30] Farkas LG, Hreczko TA. Age-related changes in selected linear and angular measurements of the craniofacial complex in healthy North American Caucasians In: Farkas LG, editor. Anthropometry of the Head and Face. second ed ed. New York: Raven Press, Ltd; 1994 p. 89–102.

[31] Yadav D, Singh R, Vatsa M, Noore A. Recognizing age-separated face images: Humans and machines. PloS one. 2014;9(12):e112234 10.1371/journal.pone.0112234 25474200 PMC4256302

